# Comparative Transcriptomics in Two Extreme Neopterans Reveals General Trends in the Evolution of Modern Insects

**DOI:** 10.1016/j.isci.2018.05.017

**Published:** 2018-05-28

**Authors:** Guillem Ylla, Maria-Dolors Piulachs, Xavier Belles

**Affiliations:** 1 Institute of Evolutionary Biology (CSIC-Universitat Pompeu Fabra), Passeig Marítim 37, 08003 Barcelona, Spain

**Keywords:** Entomology, Developmental Biology, Evolutionary Developmental Biology, Genomics

## Abstract

The success of neopteran insects, with 1 million species described, is associated with developmental innovations such as holometaboly and the evolution from short to long germband embryogenesis. To unveil the mechanisms underlining these innovations, we compared gene expression during the ontogeny of two extreme neopterans, the cockroach *Blattella germanica* (polyneopteran, hemimetabolan, and short germband species) and the fly *Drosophila melanogaster* (endopterygote, holometabolan, and long germband species). Results revealed that genes associated with metamorphosis are predominantly expressed in late nymphal stages in *B. germanica* and in the early-mid embryo in *D. melanogaster*. In *B. germanica* the maternal to zygotic transition (MZT) concentrates early in embryogenesis, when juvenile hormone factors are significantly expressed. In *D. melanogaster*, the MZT extends throughout embryogenesis, during which time juvenile hormone factors appear to be unimportant. These differences possibly reflect broad trends in the evolution of development within neopterans, related to the germband type and the metamorphosis mode.

## Highlights

• Transcriptomes of cockroaches and flies show key differences along development
• Cockroaches and flies express metamorphosis factors with distinct timings in ontogeny
• Cockroaches methylate DNA in early embryogenesis, whereas flies do not
• MZT is limited to the early embryo in cockroaches, but it extends until hatching in flies

## Graphical Abstract

**Figure:**
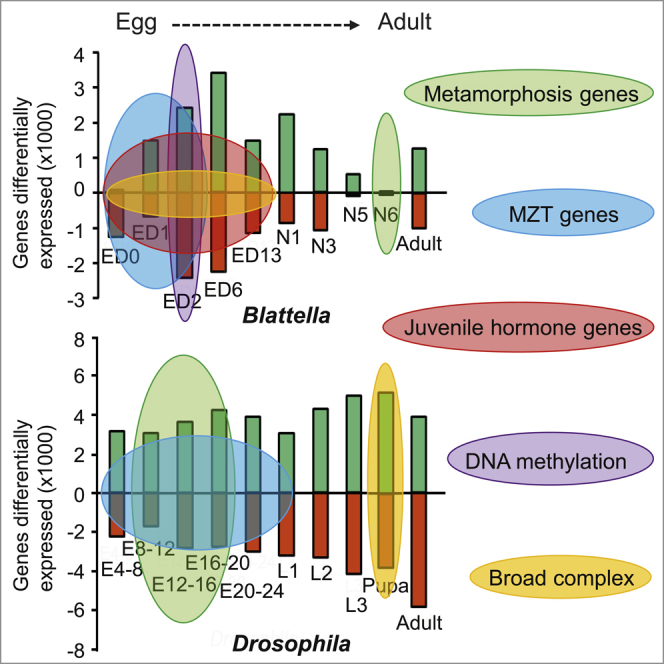

## Introduction

With around 1 million species described, insects are the most diverse animal lineage on Earth. The extraordinary success of insects is due, at least in part, to their long evolutionary history, as they emerged some 450 million years ago (Mya) (Wang et al., 2016). This gave them enough time to evolve a series of key morpho-functional innovations that acted as drivers of expansion and diversification. A crucial innovation was the acquisition of wings, which took place about 410 Mya, with the emergence of the pterygote insects (Misof et al., 2014, Wang et al., 2016). A subsequent innovation was wing flexion over the dorsal body side (thus allowing a more efficient flight), which was achieved by neopteran insects some 380 Mya (Misof et al., 2014, Wang et al., 2016). Neopterans, or modern insects, represent more than 90% of the present insect species and have colonized all major terrestrial and freshwater habitats and exploited almost every organic resource, from dead plant and animal matter to all parts of green plants, and even to other kinds of animals, as predators or parasitoids. These ecological specializations have involved the corresponding adaptations, which has led to a formidable diversity in terms of morphology, physiology, and life cycles (Grimaldi and Engel, 2005).

From a developmental point of view, another key innovation that took place within neopteran evolution was metamorphosis (Nicholson et al., 2014), by which the individual acquires characteristic adult features and stops molting during postembryonic development. The ancestral metamorphosis mode was hemimetaboly, characterized by an embryogenesis that develops a first instar nymph displaying the essential adult body structure. The nymphs grow gradually, and the final molt to the adult stage completes the formation of functional wings and genitalia (Belles, 2011). From hemimetaboly emerged a metamorphosis mode known as holometaboly, in which the embryogenesis gives rise to a larva with a body structure considerably divergent from that of the adult, often more or less vermiform. The larva grows through various stages until molting to the pupal stage, which bridges the gap between the divergent larval morphology and that of the winged and reproductively competent adult (Belles, 2011). The holometabolan mode of metamorphosis was a successful innovation, as it was accompanied by an extraordinary radiation of the insect lineage (Misof et al., 2014). Indeed, more than 80% of currently known insect species follow the holometabolan metamorphosis (Condamine et al., 2016, Grimaldi and Engel, 2005).

Parallel innovations within the neopteran history occurred in embryogenesis, such as the evolution from short to long germband development. In long germband embryogenesis, the complete body segments (head, thoracic, and abdominal segments) are configured at the blastoderm stage. In short germband embryogenesis, the head lobes and the most anterior trunk segments are configured first and new segments are subsequently added from the posterior terminus. Less modified neopteran groups, mainly polyneopterans and paraneopterans, follow the short germband embryogenesis, whereas the more modified endopterygotes follow the long germband embryogenesis, in general (Chipman, 2015, Liu and Kaufman, 2005). Another process that evolved along neopteran history is blastokinesis, the movement of the embryo into the yolk mass that usually results in a partial revolution of the embryonic body (Panfilio, 2008). Blastokinesis occurs around mid-embryogenesis and is typical of short germband, hemimetabolan insects, whereas similar movements in long germband, holometabolan species are oversimplified or practically absent (Panfilio, 2008).

Most of the information regarding the detailed mechanisms regulating development has been described in the fruit fly *Drosophila melanogaster*, the model *par excellence* for genetic studies since the end of the 19^th^ century (Markow, 2015). *D. melanogaster* is a singularly modified, endopterygote, holometabolan species, which shows long germband embryogenesis, practically without blastokinesis (Campos-Ortega and Hartenstein, 1985). The genome of *D. melanogaster* was the first to be sequenced among insects (Adams et al., 2000), but the availability of insect genomes has notably increased in recent years (I5K-Consortium, 2013). This allows entire genome comparisons, which may help to understand the genetic basis of given developmental innovations (see, for example, Harrison et al., 2018). However, developmental innovations largely evolve by altering the expression of functionally conserved genes, not by the emergence of new genes (Carroll, 2008). Therefore, comparative transcriptomics appears to be the most suitable approach to analyze the origin and evolution of developmental innovations. Again, the champion model concerning transcriptomic information is *D. melanogaster*, for which abundant high-throughput sequencing data are available, such as those generated in the modENCODE project (Celniker et al., 2009, modENCODE Consortium et al., 2010).

In contrast, transcriptomic data available in other insects are much less abundant and dispersed in a few species. Obviously, the heavy focus on *D. melanogaster* is a serious drawback if we aim at understanding the general trends of the evolution of development in insects through comparative transcriptomics. To partially fill this gap, we have produced extensive transcriptomic data along the ontogeny of the German cockroach, *Blattella germanica*, a polyneopteran hemimetabolan species, which shows short germband embryogenesis practically without blastokinesis (Tanaka, 1976), whose genome has recently been sequenced (Harrison et al., 2018). We have produced and sequenced 22 mRNA libraries from 11 developmental stages (two replicates each) covering the entire ontogeny: embryogenesis, nymphal stages, and the adult female. In total, we obtained 193,014,748 read pairs, which are now available to the scientific community. The study of these transcriptomes in *B. germanica* allowed describing the molecular basis of the main developmental transitions in this species. Then, searching in public databases, we found a most comprehensive RNA-seq dataset of *D. melanogaster* that comprises 22 libraries from 11 developmental stages (two replicates each) covering the entire ontogeny: embryogenesis, larval stages, the pupa, and the adult female (Celniker et al., 2009, modENCODE Consortium et al., 2010), with 129,507,378 read pairs in total (available at GEO: GSE18068). Then, we compared the respective ontogenetic sets of transcriptomes of *B. germanica* and *D. melanogaster*, with the idea of identifying differences among these two phylogenetically distant species that could illuminate broad trends in the evolution of development in neopteran insects.

## Results and Discussion

### General Transcriptomic and Genomic Data

In *B. germanica*, the analyses were based on 22 mRNA libraries that were prepared in our laboratory, representing the following 11 stages (two replicates each): non-fertilized egg (NFE); 8, 24, 48, 144, and 312 hr after oviposition (ED0, ED1, ED2, ED6, and ED13); first, third, fifth, and sixth (last) nymphal instars (N1, N3, N5, and N6); and adult female (Table S1). In total, we obtained 198,970,437 read pairs (data from the 22 libraries accessible at GEO: GSE99785). After removing the adapters, filtering low-quality reads with FastQC (version 0.11.4) (Andrews, 2010), and merging read pairs, we obtained 193,014,748 read pairs (corresponding to 97.0% of the total sequenced read pairs) (Table 1), 66.8% of which mapped to the *B. germanica* genome.

**Table 1:** Summary of the Reads Obtained from the Sequenced RNA-seq Libraries of *Blattella germanica*

Library	Raw Reads x2	Clean Reads x2	%	Mapped Read x2	%
NFE	14,413,472	13,736,963	95.31	11,055,040	80.48
NFE_2	12,845,235	12,715,450	98.99	9,327,382	73.35
ED0	4,664,861	4,349,066	93.23	3,348,444	76.99
ED0_2	13,374,354	13,192,165	98.64	9,187,091	69.64
ED1	2,530,147	2,339,451	92.46	1,705,713	72.91
ED1_2	9,471,555	9,027,804	95.31	6,237,668	69.09
ED2	8,023,009	7,652,728	95.38	5,603,324	73.22
ED2_2	21,825,389	20,729,119	94.98	10,263,577	49.51
ED6	8,659,285	8,427,098	97.32	6,894,844	81.82
ED6_2	10,904,515	10,667,152	97.82	6,069,177	56.90
ED13	10,364,701	10,015,047	96.63	7,892,059	78.80
ED13_2	8,523,247	8,031,716	94.23	5,656,165	70.42
N1	6,418,772	6,372,912	99.29	4,392,741	68.93
N1_2	6,429,421	6,292,351	97.87	4,062,721	64.57
N3	7,207,614	7,129,383	98.91	4,537,197	63.64
N3_2	7,564,063	7,458,733	98.61	5,230,704	70.13
N5	5,403,000	5,369,336	99.38	3,264,330	60.80
N5_2	7,655,560	7,509,961	98.10	5,012,886	66.75
N6	9,037,587	8,967,871	99.23	5,313,344	59.25
N6_2	7,237,541	7,007,056	96.82	4,024,529	57.44
Adult	8,484,768	8,412,450	99.15	5,319,340	63.23
Adult_2	7,932,341	7,610,936	95.95	4,609,493	60.56
TOTAL	198,970,437	193,014,748	96.98	129,007,769	67.66

For each library we show the number of read pairs sequenced, the number and percentage of reads after cleaning low-quality reads with Trimmomatic, and the number and percentage of clean reads mapped to the *B. germanica* genome (PRJNA427252).

The RNA-seq dataset of *D. melanogaster* used in the comparisons (GEO: GSE18068) comprises 22 libraries from 11 developmental stages (two replicates each) covering the entire embryo development (six sequential stages: 0–4 hr, 4–6 hr, 6–12 hr, 12–16 hr, 16–20 hr, 20–24 hr), the three larval stages (L1, L2, L3), the pupa, and the adult female. In postembryonic stages, we followed the correspondence *B. germanica* pre-last nymphal instars with *D. melanogaster* larvae, the last nymphal instar with the pupa (Belles and Santos, 2014), and the respective adult female stages. Correspondences between the embryo stages of *D. melanogaster* and *B. germanica* are summarized in Table S2. The analysis of the above-mentioned *D. melanogaster* libraries gave 129,507,378 read pairs, 95.2% of which mapped to the *D. melanogaster* genome (Table 2).

**Table 2:** Summary of the *Drosophila melanogaster* RNA-seq Libraries Obtained from GEO: GSE18068

Accession number	Name	Reads	Reads Mapping to the Genome	%
SRR030232	E0-4_1	3,433,652	3,292,953	95.90
SRR030233	E0-4_2	4,093,252	3,989,272	97.46
SRR030238	E4-8_1	2,822,374	2,658,024	94.18
SRR030239	E4-8_2	3,800,699	3,689,337	97.07
SRR030236	E8-12_1	5,197,055	4,947,643	95.20
SRR030237	E8-12_2	5,146,028	4,958,807	96.36
SRR030226	E12-16_1	4,908,119	4,665,060	95.05
SRR030227	E12-16_2	3,829,586	3,705,026	96.75
SRR030234	E16-20_1	9,322,851	8,456,435	90.71
SRR030235	E16-20_2	6,222,965	5,676,767	91.22
SRR030240	E20-24_1	3,488,824	3,236,901	92.78
SRR030241	E20-24_2	5,442,437	5,200,659	95.56
SRR030242	L1_1	9,611,846	8,992,150	93.55
SRR030243	L1_2	6,504,722	6,110,616	93.94
SRR030248	L2_1	9,327,073	8,888,182	95.29
SRR030249	L2_2	11,399,101	10,962,849	96.17
SRR030244	L3_1	3,667,132	3,529,505	96.25
SRR030245	L3_2	9,330,126	9,143,635	98.00
SRR030246	Pupae_1	2,616,980	2,501,962	95.60
SRR030247	Pupae_2	8,983,652	8,704,123	96.89
SRR030230	Adult-Female_1	2,143,390	2,038,572	95.11
SRR030231	Adult-Female_2	8,215,514	7,927,427	96.49
TOTAL		129,507,378	123,275,905	95.25

For each library we show the number of reads and the number and percentage of reads mapped to the *D. melanogaster* genome from Flybase (version dmel_r6.12).

We detected expression (>1 FPKM) for 90.1% of the annotated genes of *B. germanica* (25,643 out of 28,471) and 97.3% of *D. melanogaster* (17,004 out of 17,471). To facilitate comparisons, we obtained the set of orthologous genes shared by the two species. We retrieved the protein sequences from the 28,471 annotated genes of *B. germanica* and 17,471 annotated genes of *D. melanogaster* and identified 7,169 orthologous genes common to *B. germanica* and *D. melanogaster* following the best blast reciprocal hit approach. These 7,169 orthologues correspond to 25.2% of the *B. germanica* genes and 41.0% of those from *D. melanogaster*.

### General Gene Expression

**Figure 1:**
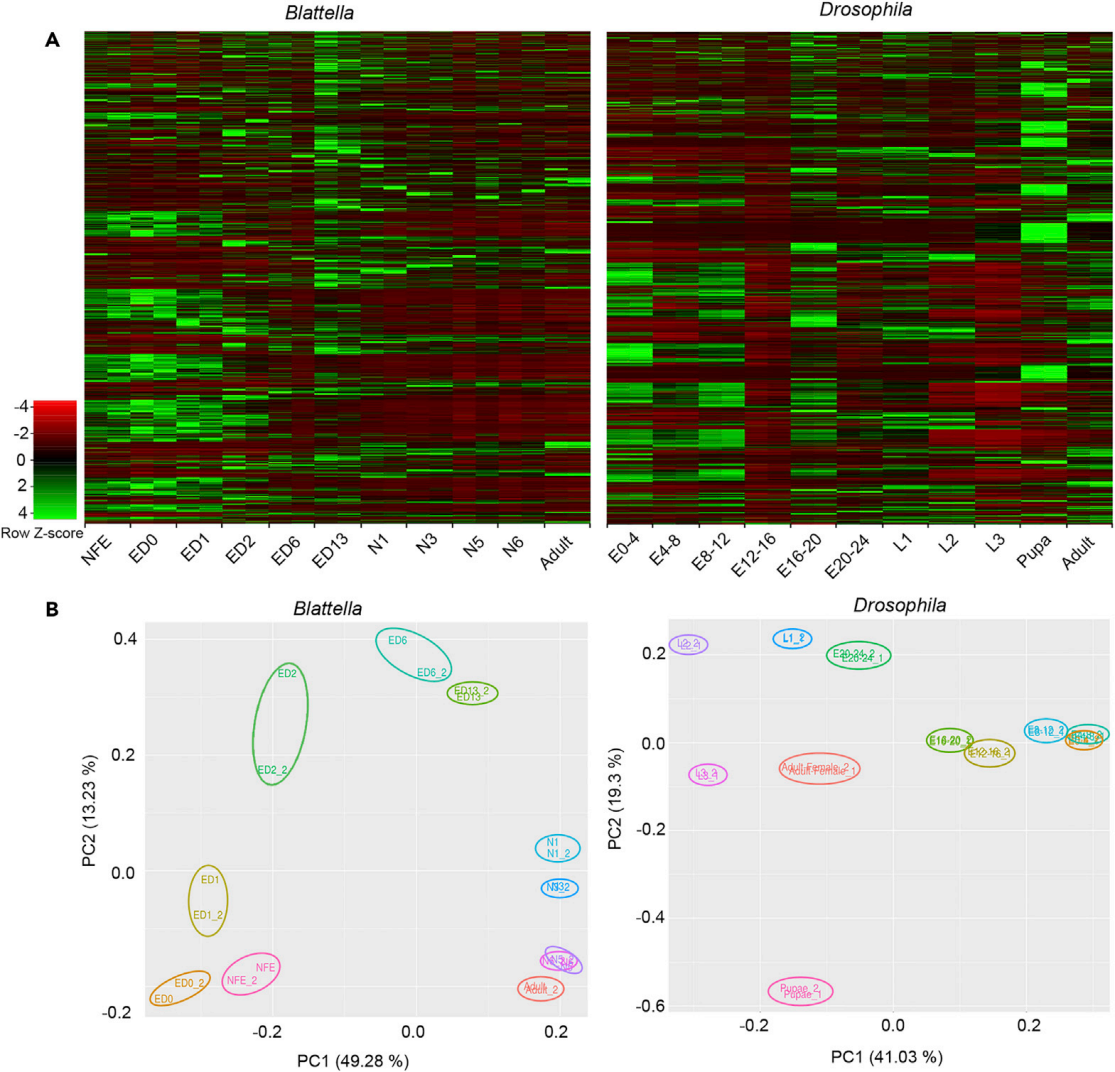
Overall Gene Expression in the Stage-Libraries of *Blattella germanica* and *Drosophila melanogaster* (A) Heatmap showing the expression of all genes (FPKM) in each of the stage-libraries. (B) Principal component analysis plot showing the distribution of the two replicates of the stage-libraries.

The expression of all genes (Figure 1A) suggests that the duplicates of each stage-library behave similarly in *B. germanica* and in *D. melanogaster*. Moreover, principal component analysis (PCA) of the expression data of all libraries indicates that the replicates of each library group together (Figure 1B), which led us to represent the replicates joined in further figures. The PCA shows that all stages are well separated from each other, except N5 and N6 in *B. germanica*, and E0-4 and E4-8 in *D. melanogaster*, which are closely related. In *B. germanica*, the general expression (Figure 1A) indicates that many genes are more abundantly expressed during embryogenesis, whereas only a relatively small set is expressed at significant amounts in postembryonic stages. In *D. melanogaster*, the distinction between embryonic and postembryonic stages in terms of the abundance of gene expression is more diffuse. Characteristically, quite a high number of genes are highly expressed in the pupa and the adult (Figure 1A).

**Figure 2:**
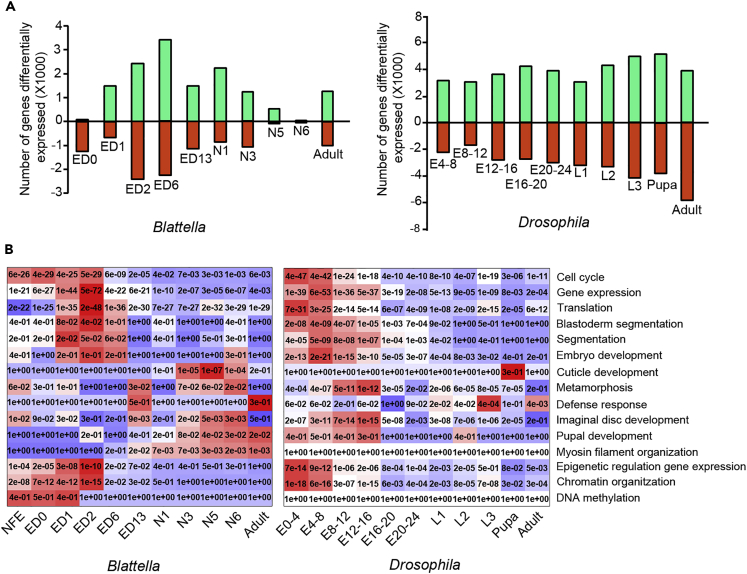
Differential Expression Analysis and GO Terms from Enrichment Analysis (A) Number of genes significantly (p < 0.05) upregulated (green) and downregulated (red) according to the differential expression analysis between consecutive stage-libraries of *Blattella germanica* and *Drosophila melanogaster*. (B) Selection of GO terms of biological processes from the enrichment analysis performed with the expressed genes at each stage in *B. germanica* and *D. melanogaster*; for each selected GO term the p value of the hypergeometric test is shown, and the color scale goes from red (low p value) to blue (high p value) normalized in each row.

In *B. germanica*, the differential expression analysis between stages reveals that the most dynamic changes occur during embryogenesis (Figure 2A). In contrast, the number of gene expression changes is maintained at similar levels in all transitions in *D. melanogaster* (Figure 2A). These differences may be related to metamorphosis, given that in the hemimetabolan mode (*B. germanica*) the basic adult body structure is formed during embryogenesis. In contrast, in holometabolan species (*D. melanogaster*) the adult morphology is completed in postembryonic stages, around the pupal stage.

The GO-terms enrichment analyses of the expressed genes reveal different biological functions at different stages within the same species and general differences between *B. germanica* and *D. melanogaster* (Figures 2B and S1). In the embryo stages, the results indicate that both species are enriched in functions related to “cell cycle control,” “gene expression,” and “translation,” suggesting an active transcriptional activity and cell proliferation, as expected in this developmental period. Functions associated with epigenetic control, such as “chromatin organization,” are also enriched, but “DNA methylation” is enriched in the early *B. germanica* embryo but not in *D. melanogaster*. Functions related to adult morphogenesis, such as “metamorphosis,” “imaginal disc development,” and “pupal development,” are enriched in the early-mid embryo in *D. melanogaster* and in late nymphal instars in *B. germanica*. This is consistent with the respective holometabolan and hemimetabolan metamorphosis mode of these species. In postembryonic development, we observed a clear enrichment in genes related to “cuticle development” in *D. melanogaster* pupae and *B. germanica* nymphs. In both species, the adult stage is enriched in genes related to homeostasis, such as metabolism, catabolism, and immune defense functions (Figure 2B).

The GO-enrichment analysis (which could suffer a bias because in *B. germanica* the GO terms are assigned on the best hit in *D. melanogaster*) is in agreement with the less informative but bias-free Pfam motifs enrichment analysis (Figure S2). Characteristically, Pfam motifs involved in metabolism and catabolism, such as those associated with peptidases, amylase, and hydrolases, and Pfam motifs related to immune defense response, such as “Defensin_2,” are enriched in genes expressed in the adult. In contrast, embryos express genes with motifs associated with the regulation of gene expression, such as Zn-finger or Homeobox genes.

### Genes Mainly Associated with Embryogenesis

We paid special attention to maternally loaded transcripts, genes involved in the maternal to zygotic transition (MZT), genes driving the early embryo patterning, and Hox genes.

The NFE libraries of *B. germanica* contain maternally loaded mRNAs enriched for functions related to “cell cycle” and “embryo development” (Figures 2B and S1), as could be expected. They are also enriched for epigenetic functions (“epigenetic regulation of gene expression,” “DNA methylation,” and “chromatin organization”), but these GO terms do not appear in the earliest stage-libraries of *D. melanogaster* (Figures 2B and S1). Especially intriguing are the genes with the GO terms “metamorphosis” and “wing disc development” occurring in the NFE library of *B. germanica*. These include genes involved in the formation of bristles (*hairless*, *spineless*), legs (*croocked legs*, *rotund, spineless*, *vulcan*), antennae (*rotund*, *spineless*), and compound eyes (*Tartan*, *Hyperplastic discs*, *eyes absent*, *rotund*). The function of maternal transcripts with these GO terms is enigmatic but might be related to the hemimetabolan metamorphosis of *B. germanica*.

**Figure 3:**
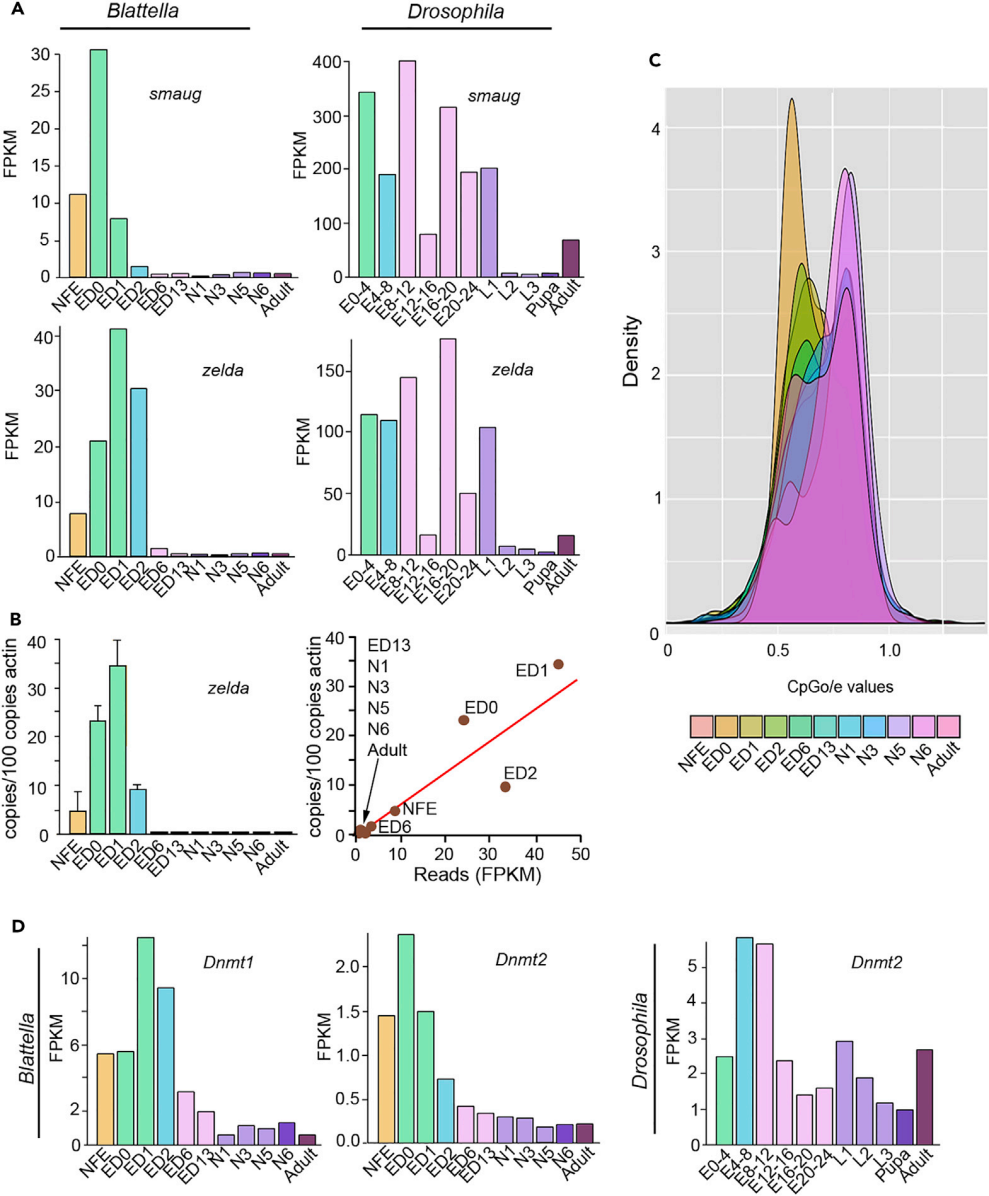
Gene Expression and Methylation during the Maternal to Zygotic Transition in *Blattella germanica* and *Drosophila melanogaster* (A) Reads-based expression of *smaug* and *zelda* along the different stage-libraries. (B) Left: qRT-PCR-based expression of *zelda* along the same stages in *B. germanica*; each value represents three biological replicates and it is represented as copies of *zelda* mRNA per 100 copies of BgActin-5c mRNA (mean ± SEM). Right: Representation of the FPKM and qRT-PCR values of expression of *zelda* in the stages studied and the regression line obtained. (C) The CpGo/e distribution of the differentially expressed genes in each stage-library of *B. germanica*. (D) Expression of DNA methyltransferase *Dnmt1* and the tRNA methyltransferase *Dnmt2*, along the different stage-libraries. In *A*, *B*, and *D*, identical bar colors indicate equivalent developmental periods, according to the criteria summarized in Table S2.

In the MZT transition of *D. melanogaster*, important genes are *smaug* (*smg*), associated with the elimination of maternal transcripts (Benoit et al., 2009, Chen et al., 2014, Tadros et al., 2007), and *zelda* (*zld*), involved in the activation of the zygotic genome (Foo et al., 2014, Liang et al., 2008, Nien et al., 2011, Schulz et al., 2015, Sun et al., 2015). Moreover, Zelda promotes the expression of Mir-309 microRNAs (Fu et al., 2014) that, in turn, contribute to eliminating maternal mRNAs (Bushati et al., 2008). In *B. germanica*, *smg* shows an expression peak in ED0, whereas *zld* peaks in ED1, in both cases followed by an abrupt expression decrease, keeping low values during the remaining ontogeny. In contrast, *smg* and *zld* are consistently expressed during all embryogenesis and in the first larval instar of *D. melanogaster* (Figure 3A). In *D. melanogaster*, *smg* expression has been studied in terms of protein by western blot along the embryogenesis by Smibert et al. (1999), who observed a signal only in the first 3 hr of embryo development. However, the signal shown is very tenuous, which casts doubts about the possibility that a higher protein load would have allowed detecting signal at late embryogenesis. Subsequent works present *smg* western blot analyses only for the first 3–4 hr of embryogenesis (Benoit et al., 2009, Dahanukar et al., 1999). Regarding *zld* in *D. melanogaster*, northern blot analyses had shown that expression appears to be quite high in the embryo, L1 and L2, then decreases in L3 and the pupa, and slightly increases in the adult (Staudt et al., 2006), which is fairly coincident with the reads-based pattern obtained by us (Figure 3A). In *B. germanica* we sought to validate the reads-based pattern with qRT-PCR measurements. Interestingly, the obtained qRT-PCR profile showed a strong and significant correlation with the reads-based pattern (Pearson correlation of 0.904 with a p value = 0.00013) (Figure 3B).

In *B. germanica*, the expression of *smg* is compatible with the role of eliminating maternal transcripts and that of *zld* is compatible with a stimulatory role on Mir-309 microRNAs expression, which, according to Ylla et al. (2017), peaks on ED2. In *B. germanica*, *smg*, *zld*, and Mir-309 show a narrow window of expression between ED0 and ED2, framing the MZT within the first 12% of embryogenesis. In *D. melanogaster*, *smg* and *zld* maintain quite high levels of expression throughout embryogenesis and even the first larval instar (Figure 3A). This continued expression of *smg* and *zld* in *D. melanogaster* that look like an “extended” MZT, is consistent with the stable expression changes along all embryogenesis (Figure 2A), and might be related to the formation of the morphologically divergent holometabolan larva.

The functional enrichment analysis (Figure 2B) suggested that DNA methylation operates during the MZT in *B. germanica* embryos, whereas this is not the case with *D. melanogaster*. Thus, we examined the CpG depletion (CpGo/e, observed versus expected number of CpGs), which is a reliable predictor of DNA methylation (Bewick et al., 2016). The comparison of CpGo/e with gene expression in the 11 stage-libraries of *B. germanica* revealed a significant negative correlation between both parameters in ED0, ED1, and ED2 stages. The genes overexpressed in these stages, covering the MZT, had the lowest levels of CpGo/e (Figure 3C). Moreover, *Dnmt1*, a gene coding a DNA methyltransferase (Lyko, 2018), is predominantly expressed in these same stages (Figure 3D). Interestingly, the expression of *Dnmt2*, whose gene product catalyzes tRNA methylation (Goll et al., 2006), also peaks in very early embryo development (Figure 3D). In *D. melanogaster*, the expression of *Dnmt2* shows a peak around mid-embryogenesis and then a significant expression is kept all along ontogeny (Figure 3D), a pattern that is in agreement with previous northern and western blot studies (Kunert et al., 2003, Lyko et al., 2000). *D. melanogaster* does not have *Dnmt1*, which is consistent with data suggesting that DNA methylation is quantitatively irrelevant in dipterans (Marhold et al., 2004), although limited DNA methylation has been observed to occur in short motifs, independent of Dnmt2 (Takayama et al., 2014).

Our data suggest that a discrete wave of DNA methylation promotes temporal expression of a set of genes during the MZT of *B. germanica* that might be necessary for the zygotic activation. DNA methylation is currently associated with a repressed chromatin state and inhibition of gene expression, although in some instances it can also have an activating effect (Siegfried and Simon, 2010). In insects, levels of DNA methylation are much higher in the hemimetabolan than in the holometabolan species (Bewick et al., 2016), although DNA methylation appears to be important for caste differentiation in holometabolan, social bees (Elango et al., 2009), and ants (Kay et al., 2018), as well as in hemimetabolan termites (Harrison et al., 2018). Among the holometabolan groups, DNA methylation is limited and atypical in *D. melanogaster* (Dnmt1- and Dnmt3-independent) (Elango et al., 2009, Marhold et al., 2004, Takayama et al., 2014). In the beetle *Tribolium castaneum*, which possesses Dnmt1 and Dnmt2, but not Dnmt3, two types of DNA methylation exist: conserved CpG methylation catalyzed by Dnmt1 and non-CpG methylation, which shows high similarity to *D. melanogaster* methylation and would be catalyzed by still unknown methyltransferases (Feliciello et al., 2013, Song et al., 2017). The contrast between the high and low levels of DNA methylation in hemimetabolan and holometabolan species, respectively (Bewick et al., 2016), and our observations in *B. germanica* suggest that DNA methylation operates in early embryo development of hemimetabolan species, contributing to the zygote gene activation in the MZT. We propose that this is an ancestral feature in neopteran insects, whose functional relevance may have been progressively lost in holometabolan species (see also Bewick et al., 2016).

**Figure 4:**
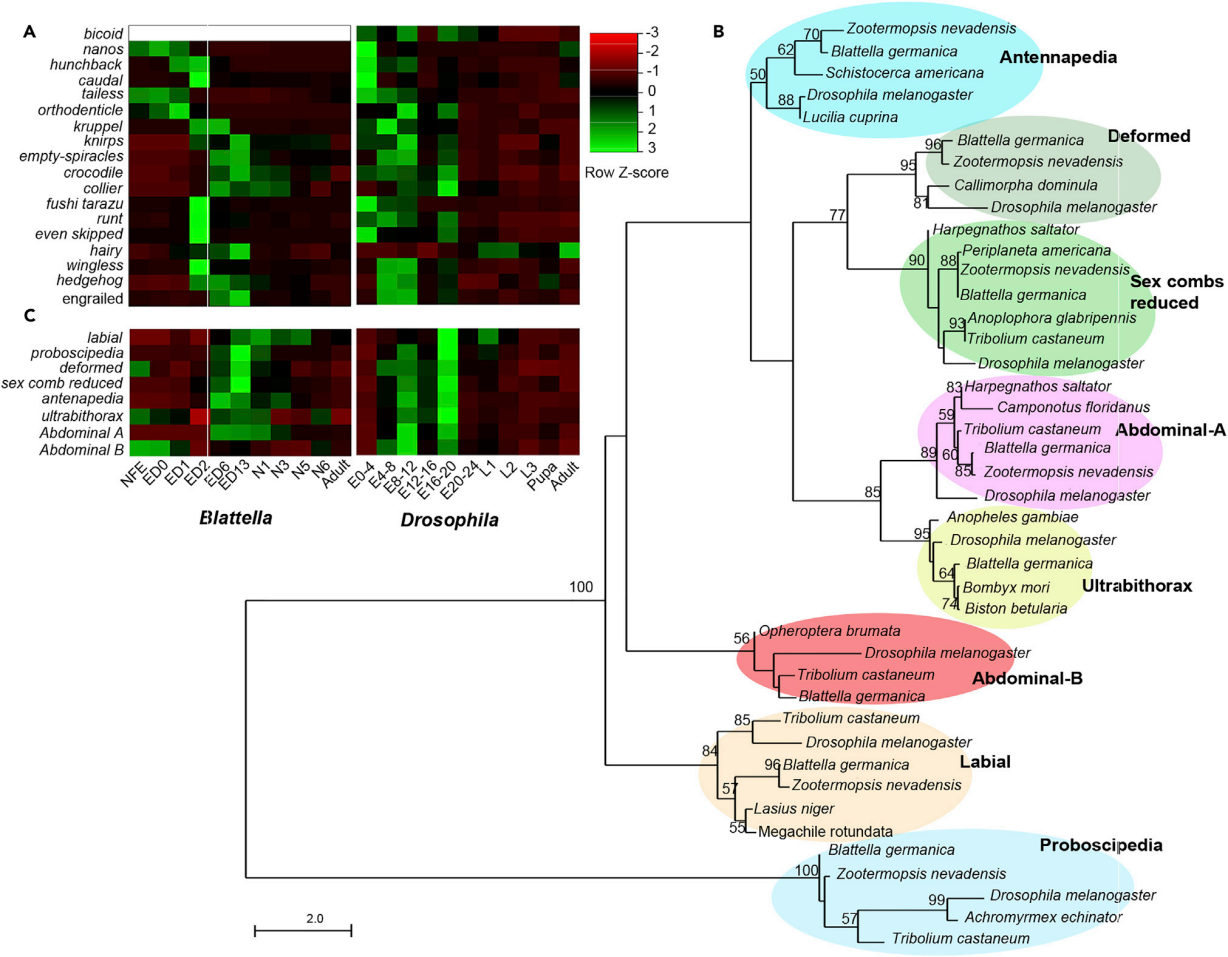
Expression of Early Patterning Genes, Hox Genes, Transcription Factors (TFs), and Genes Related to Hormonal Biosynthesis and Signaling along the Different Stage-Libraries of *Blattella germanica* and *Drosophila melanogaster* (A) Heatmap showing the expression of maternal, gap, pair-rule, and segmentation genes; *bicoid* has no orthologs in *B. germanica*. (B) Phylogenetic relationships of the Hox proteins of *Blattella germanica* with those of other insect species. Bootstrap values > 50 are indicated in the corresponding nodes. Scale bar indicates the number of substitutions per site. (C) Heatmap showing the expression of Hox genes. In (A) and (C), the expression is indicated in FPKM.

Concerning early embryo patterning, we examined the expression of the most representative gap, pair-rule, and segment polarity genes, which determine the general polarity of the embryo (Peel et al., 2005). The most obvious difference between *B. germanica* and *D. melanogaster* in very early embryogenesis is the absence of *bicoid* in the former species, as this gene is exclusive to higher dipterans (Schröder, 2003). In *B. germanica*, the gap-gene cascade is initiated by maternal *tailless*, followed by *orthodenticle*, *huckbein*, and *Krüppel* (Figure 4A). In general, the expression patterns are similar in both species, showing approximately the cascade of maternal, gap, pair-rule, and segment polarity genes. Only *hairy* (*h*) exhibits a neatly divergent pattern, being predominantly expressed in mid-late embryogenesis in *B. germanica* and in postembryonic stages in *D. melanogaster* (Figure 4A). In *D. melanogaster*, *h* acts as a pair-rule in early embryo development, whereas in larvae, by binding to the protein Achaetae, regulates the patterning of sensory organs in the developing wings and legs (Fisher and Caudy, 1998). Through other mechanisms, *h* might also contribute to regulating the morphogenetic furrow in the developing eye (Bhattacharya and Baker, 2012). The latter functions explain the expression that we observed in *D. melanogaster* larvae, and we speculate that the high level of expression in the mid-late embryo of *B. germanica* might be due to the formation of nymphal structures, such as proper chaetotaxy and compound eyes.

Subsequently, we examined the Hox genes, which play key roles in morphogenesis and body structure shaping (Averof and Akam, 1995). We identified the eight canonical Hox genes in the *B. germanica* genome (Figure 4B) and observed that most of them are fully expressed in the mid-late embryo, such as in *D. melanogaster*, when dorsal closure occurs (Figure 4C). The main difference between the two species is *Abdominal-B* (*Abd-B*), which, in *B. germanica*, shows the highest transcript levels in NFE and ED0. The function of the high maternal load of *Abd-B* is enigmatic, but the low expression levels in the mid-late embryo might have to do with dorsal closure. In *D. melanogaster*, mixer cell remodeling regulates tension along the leading edge during dorsal closure. *Abdominal-A* (*abd-A*) is a pro-mixing factor in the first five abdominal segments, whereas *Abd-B* represses mixing in posterior segments. At late closure in the central segments, the tension increases and *abd-A* is not repressed by *Abd-B* in these segments (Roumengous et al., 2017). If *abd-A* and *Abd-B* played the same role in *B. germanica*, then the low expression of *Abd-B* would suggest that the pro-mixing action of *abd-A* is needed all along the leading edge during dorsal closure.

### Transcription Factors

**Figure 5:**
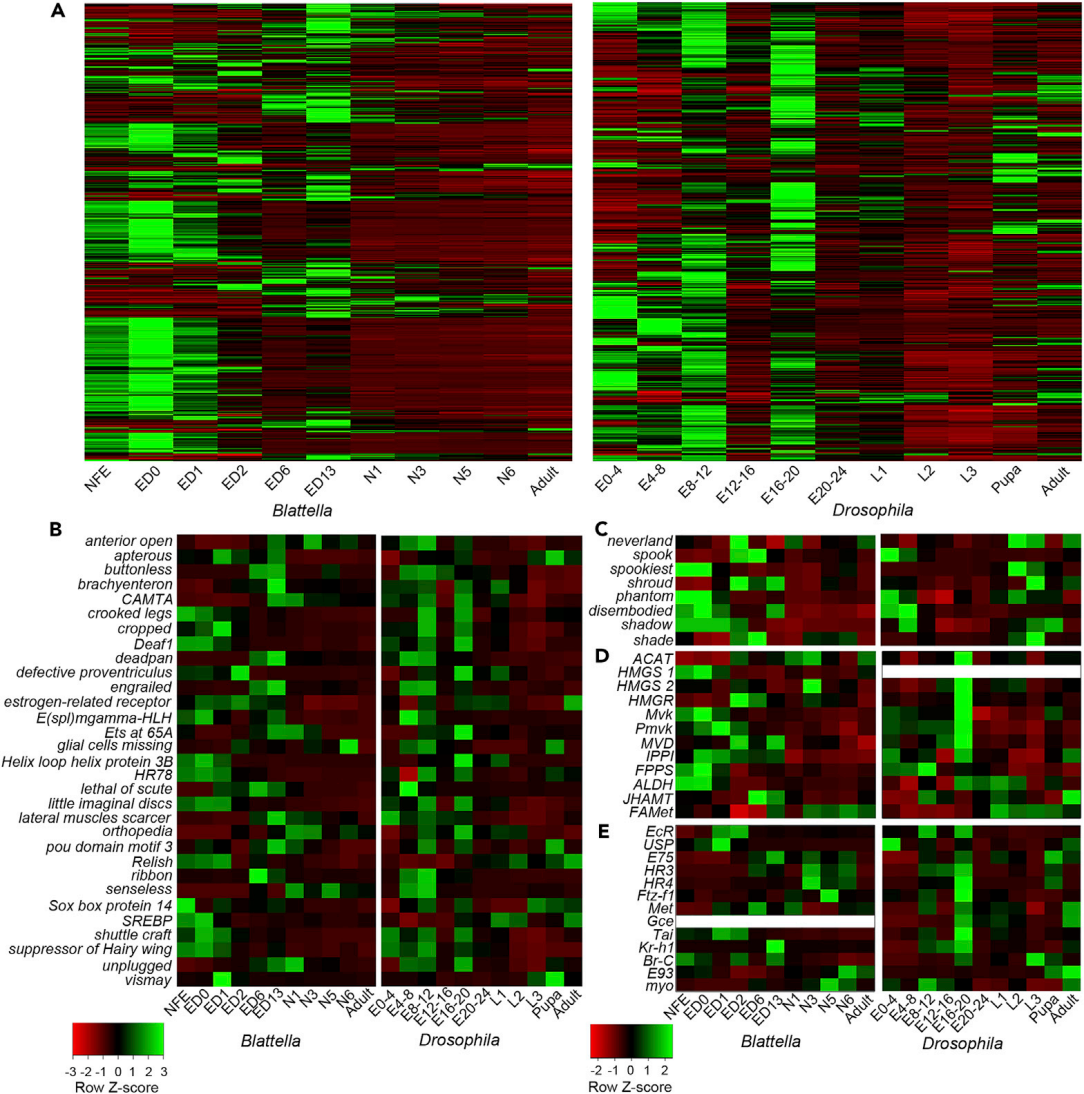
Expression of Transcription Factors (TFs) and Genes Related to Hormonal Biosynthesis and Signaling along the Different Stage-Libraries of *Blattella germanica* and *Drosophila melanogaster* (A) Expression of genes containing at least one Pfam motif unequivocally linked to a transcription factor function in *B. germanica* (600 genes identified) and in *D. melanogaster* (458 genes identified). (B) Selection of 34 orthologous TFs common to *B. germanica* and *D. melanogaster* with conspicuously divergent expression. (C) Genes coding for enzymes for ecdysone (20E) synthesis. (D) Genes coding for enzymes for juvenile hormone (JH) synthesis; *HMGS1* has no orthologs in *D. melanogaster*. (E) Genes coding for key TFs that transduce the 20E and JH signals; *gce* and *Met* have only one ortholog in *B. germanica*. The expression is shown in FPKM in all cases. The left color scale refers to panels (A) and (B), and the right one to panels (C), (D) and (E).

Important players in gene regulatory networks are transcription factors (TFs) (de Mendoza et al., 2013). To study them, we performed a PfamScan search in annotated proteins, which gave 17,196 PFAM-A motifs (4,280 unique) associated with 12,789 *B. germanica* genes and 15,475 (4,339 unique) associated with 10,759 *D. melanogaster* genes. Among these, we identified 600 genes in *B. germanica* and 458 in *D. melanogaster* that contained at least one Pfam motif associated with a TF function (de Mendoza et al., 2013, Ylla and Belles, 2015). Most of these TFs are differentially expressed during embryogenesis of both species, and many of them are also highly expressed in the pupal and adult stages of *D. melanogaster* (Figure 5A). To identify comparable differences between the two species, we retrieved the subset of orthologous TF genes common to *B. germanica* and *D. melanogaster*, obtaining 297 genes shared by the two species (Data S1). The expression of these 297 genes in *B. germanica* and *D. melanogaster* (Figure S3) reminds that observed when representing all genes (Figure 1A) or all TFs (Figure 5A), with many TF genes more abundantly expressed during embryogenesis in *B. germanica*, whereas in *D. melanogaster* the diversity of expression is more similar in embryonic and postembryonic stages.

A selection of TF genes that display a greater contrast in expression between species and stages is shown in Figure 5B. We can see, for example, that *vismay*, *SREBP* (sterol regulatory element binding protein), and *HLH3B* (helix loop helix protein 3B) are specifically highly expressed in the very early embryonic stages (ED0 and ED1) of *B. germanica*. High expression of *SREBP* suggests that lipogenesis and lipid homeostasis (Shao and Espenshade, 2012) are important in these stages in *B. germanica*. Conversely, *SREBP* appears to be not as relevant in *D. melanogaster*, but others, such as *lateral muscles scarcer*, involved in the development of embryonic lateral transverse muscles (Müller et al., 2010), are highly expressed in early embryo development. In mid-late embryogenesis, *lethal of scute*, a gene involved in the neurogenesis and specification of sensory organs (Negre and Simpson, 2015), is highly expressed in *B. germanica*, whereas *shuttle craft*, required to maintain the proper morphology of motoneuronal axon nerve routes (Stroumbakis et al., 1996), is highly expressed in *D. melanogaster*. Also typical of late embryo stages of *D. melanogaster* is the high level of expression of *little imaginal discs*, a histone demethylase that specifically removes H3K4me3, a mark associated with active transcription (Li et al., 2010), and *cropped*, a gene essential for embryonic tracheal terminal branching (Wong et al., 2015). The aforementioned expression divergences refer to genes with no relevant roles in general patterning or organogenesis and could respond to circuitries specific of *B. germanica* and *D. melanogaster*, rather than being considered as reflecting general trends of evolution of development.

With respect to postembryonic stages, *unplugged* (*unpg*), required for the formation of specific tracheal branches (Chiang et al., 1995), and *senseless* (*sens*), crucial for the peripheral nervous system development (Nolo et al., 2000), are typically highly expressed in *B. germanica* nymphs, whereas in *D. melanogaster*, expression of these genes appears to be insignificant. Conversely, *Sox14,* required for 20E signaling at the onset of metamorphosis (Ritter and Beckstead, 2010), and *Relish* (*Rel*), which promotes the transcription of innate immune response genes (Petersen et al., 2013), are characteristically expressed in *D. melanogaster* larvae. The pupa of *D. melanogaster* continues expressing *Sox14* and *Rel* at high levels. These expression divergences may reflect the different development of cockroach nymphs and fly larvae and pupae. Compared with fly larval growth, the development of cockroach nymphs involves a considerable increase in size; thus, growth of the tracheal and the peripheral nervous systems promoted by *unpg* and *sens* makes sense in this context. The expression of *Sox14* in larvae and pupae might be related to the complex ecdysone signaling that regulates the holometabolan postembryonic development, which requires precise increases and decreases of hormonal signaling in narrow temporal windows (Riddiford et al., 2003). The continued expression of *Rel* in the pupa must be associated with the vulnerability to infections of this immobile stage.

### Genes Associated with Metamorphosis

We have considered genes related to the two main hormones regulating metamorphosis, the juvenile hormone (JH) and the ecdysone, or more properly 20-hydroxyecdysone (20E), which is the most well-known active form. During the juvenile postembryonic life, JH levels are high, but in the pre-adult stage, they fall dramatically until being practically undetectable. JH has a repressor role upon metamorphosis, and its absence determines the metamorphosis. 20E has an ecdysteroidal structure, and during juvenile stages, it is synthesized by the prothoracic glands. The most important role of 20E is to promote molting, and in the pre-adult stage, in the absence of JH, it promotes the metamorphic molt (Belles, 2011, Nijhout, 1994).

In general, the genes involved in 20E biosynthesis are more highly expressed in all embryonic stages than in nymphs or adults in *B. germanica*, whereas in *D. melanogaster* they are mostly expressed in very early embryos and pre-adult stages (Figure 5C). The genes involved in JH biosynthesis are expressed throughout the ontogeny of *B. germanica* and *D. melanogaster* in a relatively similar way, although in the very early embryogenesis expression is high in *B. germanica* and low in *D. melanogaster* (Figure 5D), whereas only in late embryo stages, especially in the E16-20 stage, it becomes high in *D. melanogaster*. This appears to be the general trend: JH (and JH signaling) appears earlier in hemimetabolan than in holometabolan species (Truman and Riddiford, 1999). Moreover, the high expression of JH genes in early embryogenesis observed in *B. germanica* may be typical of the hemimetabolan species. Indeed, JH genes have been shown to have important functions in early embryogenesis of *B. germanica* (Fernandez-Nicolas and Belles, 2017), which does not appear to be the case in the holometabolan silkworm, *Bombyx mori* (Daimon et al., 2015).

In *D. melanogaster*, the expression of typical transducers of the 20E signal (King-Jones and Thummel, 2005), such as *ecdysone receptor* (*EcR*), *ultraspiracle* (*USP*), *E75*, *HR3*, and *HR4*, appears to be more concentrated in the E16-20 stage, whereas in *B. germanica* it spreads in earlier embryo stages. Singularly, *Fushi tarazu factor 1* (*Ftz-f1*) exhibits a predominant expression in N5 in *B. germanica*, whereas in *D. melanogaster* it is mainly expressed in mid-embryogenesis (Figure 5E). The characteristic expression in *B. germanica* may suggest that *Ftz-f1* plays important roles in the penultimate nymphal instar, when it is defined the genetic program of the last nymph (in which metamorphosis is determined). We have reported previously that *Ftz-f1* has critical functions during the last nymphal molts in *B. germanica* (Cruz et al., 2008). Concerning JH transducers (Jindra et al., 2015), there are not great differences of expression patterns of *Methoprene-tolerant* (*Met*), *Taiman* (*Tai*), and *Krüppel homolog 1* (*Kr-h1*) between the two species studied (Figure 5E). *Broad-complex* (*BR-C*) is interesting, as its expression shows a divergent pattern in *D. melanogaster*, where it is concentrated in the last larval instar and the pupa. In *B. germanica* maternal *BR-C* transcripts are abundant, and the gene is significantly expressed during embryogenesis (Figure 5E). This is consistent with the important functions of *BR-C* in embryo development of this species (Piulachs et al., 2010), whereas in postembryonic development *BR-C* is involved in promoting wing pad growth (Huang et al., 2013). Conversely, *BR-C* has a key function in pupal morphogenesis in *D. melanogaster* and in holometabolan insects, in general (Zhou and Riddiford, 2002). The occurrence of significant amounts of *BR-C* transcripts in the maternal load of *B. germanica* could be associated with the formation of the short germband type of this species, whereas their expression in mid and late embryogenesis might be related to the formation of a first instar nymph with basic adult features, typical of the hemimetabolan mode of metamorphosis. Other differences in the expression of JH-associated genes between *B. germanica* and *D. melanogaster* during postembryonic development may be simply idiosyncratic, as JH does not completely repress metamorphosis in higher flies, as occurs generally in insects, including cockroaches (Riddiford and Ashburner, 1991).

Other genes related to metamorphosis are *E93*, which triggers adult morphogenesis in hemimetabolan and holometabolan species (Belles and Santos, 2014, Ureña et al., 2014), and *myoglianin* (*myo*), which in the cricket *Gryllus bimaculatus* regulates the JH decrease that occurs in the last nymphal instar, which triggers metamorphosis (Ishimaru et al., 2016). Concentrated *E93* expression in pre-adult and adult stages in both *B. germanica* and *D. melanogaster* (Figure 5E) is consistent with its role of adult specifier. The high expression of *myo* in the pre-adult stages of *B. germanica* (Figure 5E) is in agreement with the inhibitory role on JH production described in *G. bimaculatus*. This role could be, therefore, conserved in hemimetabolan species, but not in holometabolan species, such as *D. melanogaster*, where *myo* expression is practically absent in pre-adult stages but concentrates in mid embryogenesis (Figure 5E), which is consistent with its role in the formation of embryo glial cells and myoblasts (Lo and Frasch, 1999).

### Conclusions

A significant part of the transcriptomic differences observed appears to be specific of cockroaches or flies. This must be the case of expression divergences in many TFs, which probably reflect differences in the expression and circuitry in functionally similar genetic networks. However, the differences underlined later might reflect broad trends in the evolution of basic processes within neopterans, such as the development of the germband type or the metamorphosis mode.

*B. germanica* exhibits the most dynamic gene expression changes during embryogenesis. In contrast, *D. melanogaster* keeps a similar level of expression changes throughout ontogeny. This may be related to the different types of metamorphosis: hemimetabolan in cockroaches, where the adult body structure is shaped during embryogenesis, and holometabolan in flies, which shapes the adult morphology in postembryonic stages. Genes associated with adult morphogenesis (“metamorphosis,” “imaginal disc development,” “pupal development”) are predominantly expressed in late nymph stages in *B. germanica* and in the early-mid embryo in *D. melanogaster*. Again, this reflects a basic difference between hemimetabolan and holometabolan metamorphosis.

In *B. germanica*, the expression of *smg* and *zld*, which are important players in the MZT (see Liang et al., 2008, and Nien et al., 2011, for functional studies), concentrates in early embryogenesis (from 0% to 12% development), whereas in *D. melanogaster* there is significant expression throughout the entire embryogenesis. This sort of “extended” MZT might be related to the evolutionarily derived embryo morphogenesis and to the hemimetabolan mode of metamorphosis.

DNA methylation in early embryogenesis, which possibly promotes the expression of genes involved in the zygotic activation, is detected in *B. germanica* but not in *D. melanogaster*. This is consistent with the fact that hemimetabolan species have high levels of DNA methylation, in general, whereas they are much lower in holometabolans (Bewick et al., 2016). Thus, progressive loss of DNA methylation, in this case in the embryo, may have been a mechanism driving the evolution from hemimetabolan polyneopterans and paraneopterans to holometabolan endopterygotes.

The expression of TFs reveals many differences between *B. germanica* and *D. melanogaster* in embryonic and postembryonic stages. Many of them appear to be specific, but some observed in JH and 20E transducers could be representative of the type of embryogenesis and/or metamorphosis. For example, transcripts of JH transducers that are present at significant amounts in very early embryo stages of *B. germanica*, but not in *D. melanogaster*, may reflect a different regulation of the blastoderm formation related to the germband type, short (*B. germanica*) or long (*D. melanogaster*) (see Fernandez-Nicolas and Belles, 2017, for functional studies). It is plausible that loss of these JH transducers in the very early embryo has been one of the drivers of evolution from short to long germband. Another difference relates to the expression of these hormonal transducers in the mid and late embryo, which is quantitatively and functionally important in hemimetabolan species (Fernandez-Nicolas and Belles, 2017, Piulachs et al., 2010) but not in holometabolans (Daimon et al., 2015). Thus, the declining influence of JH in the embryo may have been another factor driving the morphological divergence of juvenile stages in the holometabolan last common ancestor and the evolution of metamorphosis toward holometaboly.

Comparisons also highlighted *BR-C* as a particularly important TF. In *D. melanogaster*, *BR-C* expression concentrates in prepupal and pupal stages, which is consistent with its key role in pupal morphogenesis of holometabolan insects (Zhou and Riddiford, 2002). In turn, the low expression during embryogenesis fits with the practically dispensable role of *BR-C* in embryo development in holometabolan insects (Daimon et al., 2015). In *B. germanica*, in contrast, the highest expression of *BR-C* is observed along embryogenesis, which is in agreement with its important morphogenetic roles (Piulachs et al., 2010), which would be characteristic of embryogenesis in hemimetabolan species. Comparatively, the expression of *BR-C* in nymphal stages is low, which corresponds to its limited role of sustaining the growth of wing pads (Huang et al., 2013). As proposed by Huang et al. (2013), a fundamental innovation in postembryonic development in holometabolans has been an expansion of *BR-C* functions, from one specialized in wing development to a larger array of morphogenetic functions that culminated with the pupal specifier role. Conversely, in hemimetabolans, *BR-C* would have important morphogenetic roles in embryo development, and its loss may have been an important factor in the evolution of holometaboly from hemimetaboly (see also Fernandez-Nicolas and Belles, 2017).

## Methods

All methods can be found in the accompanying Transparent Methods supplemental file.

## Supplemental Information

Document S1. Transparent Methods, Figures S1–S3, and Tables S1–S3

Data S1. Transcription Factors Common to *Drosophila melanogaster* (Dmel) and *Blattella germanica* (Bger) and Expression in Each Stage-Library, Related to Figure 5A

Data S2. Table of Counts for the *Blattella germanica* Libraries, Related to Transparent Methods

Data S3. Table of Counts for the *Drosophila melanogaster* Libraries, Related to Transparent Methods

Data S4. Gene IDs from the Orthologous Genes Identified by Blast Reciprocal Best Hit (BRBH) between *Blattella germanica* and *Drosophila melanogaster*, Related to Transparent Methods The gene symbol and gene name correspond to that of *D. melanogaster*.
