# Supplementary material for: Comparative Transcriptomics in Two Extreme Neopterans Reveals General Trends in the Evolution of Modern Insects
Source: iScience. 2018 May 28;4:164–79. doi: 10.1016/j.isci.2018.05.017 (PMC6147021; doi:10.1016/j.isci.2018.05.017)
Supplement: Document S1. Transparent Methods, Figures S1–S3, and Tables S1–S3 [file mmc1.pdf]

ISCI, Volume *4*

Supplemental Information

Comparative Transcriptomics in Two Extreme Neopterans Reveals General Trends in the Evolution of Modern Insects

Guillem Ylla, Maria-Dolors Piulachs, and Xavier Belles

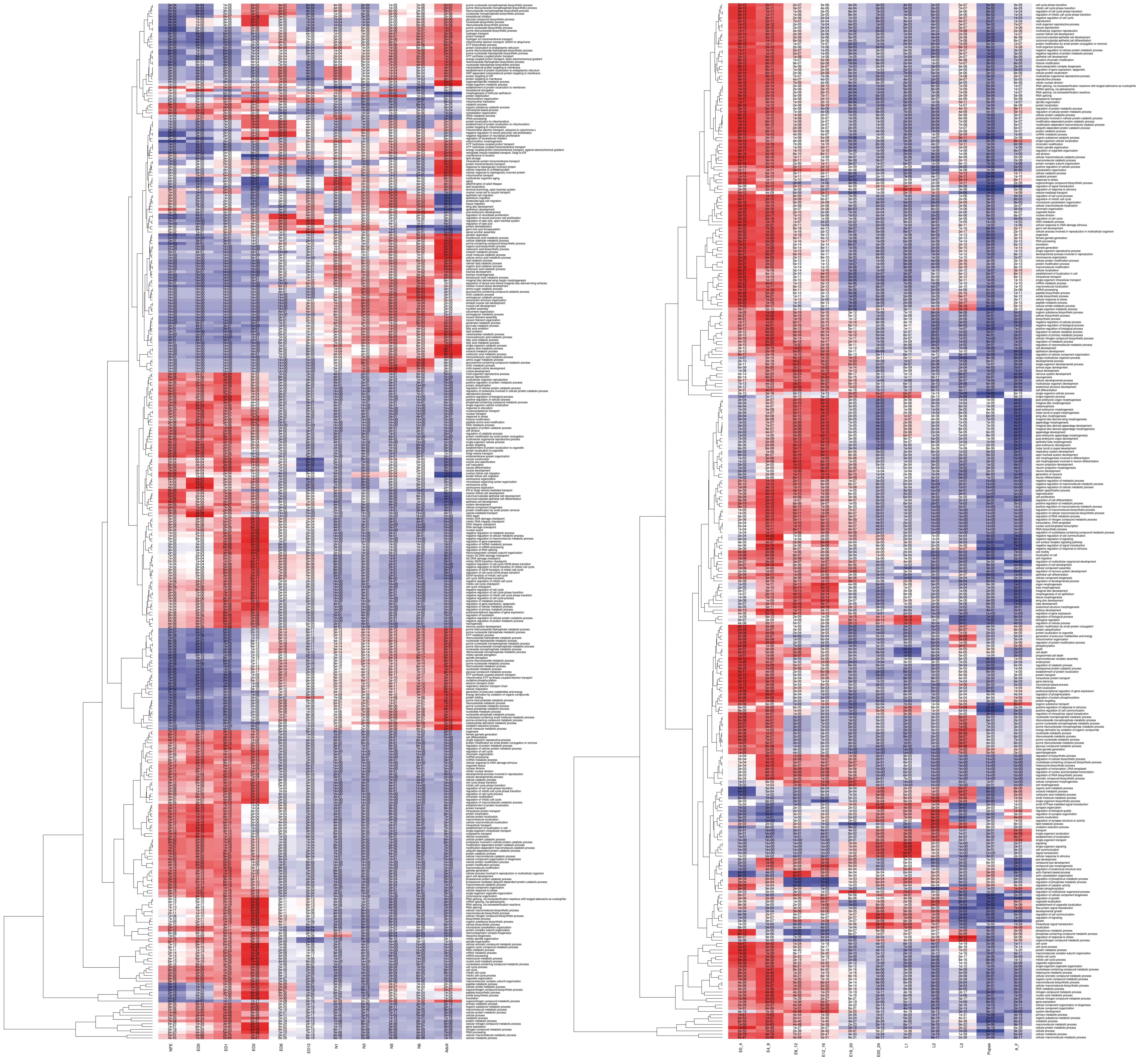

Figure S1. Biological process GO-terms enrichment analysis carried out in each stage-library of *Blattella germanica* (left) and *Drosophila melanogaster* (right). Related to Figure 2B. Only genes whose expression was >1 FPKM were considered. For each GO-term at each library, the p-value of the hypergeometric test is shown. The color scale from red (low p-value) to blue (high p-value) is normalized at each row.

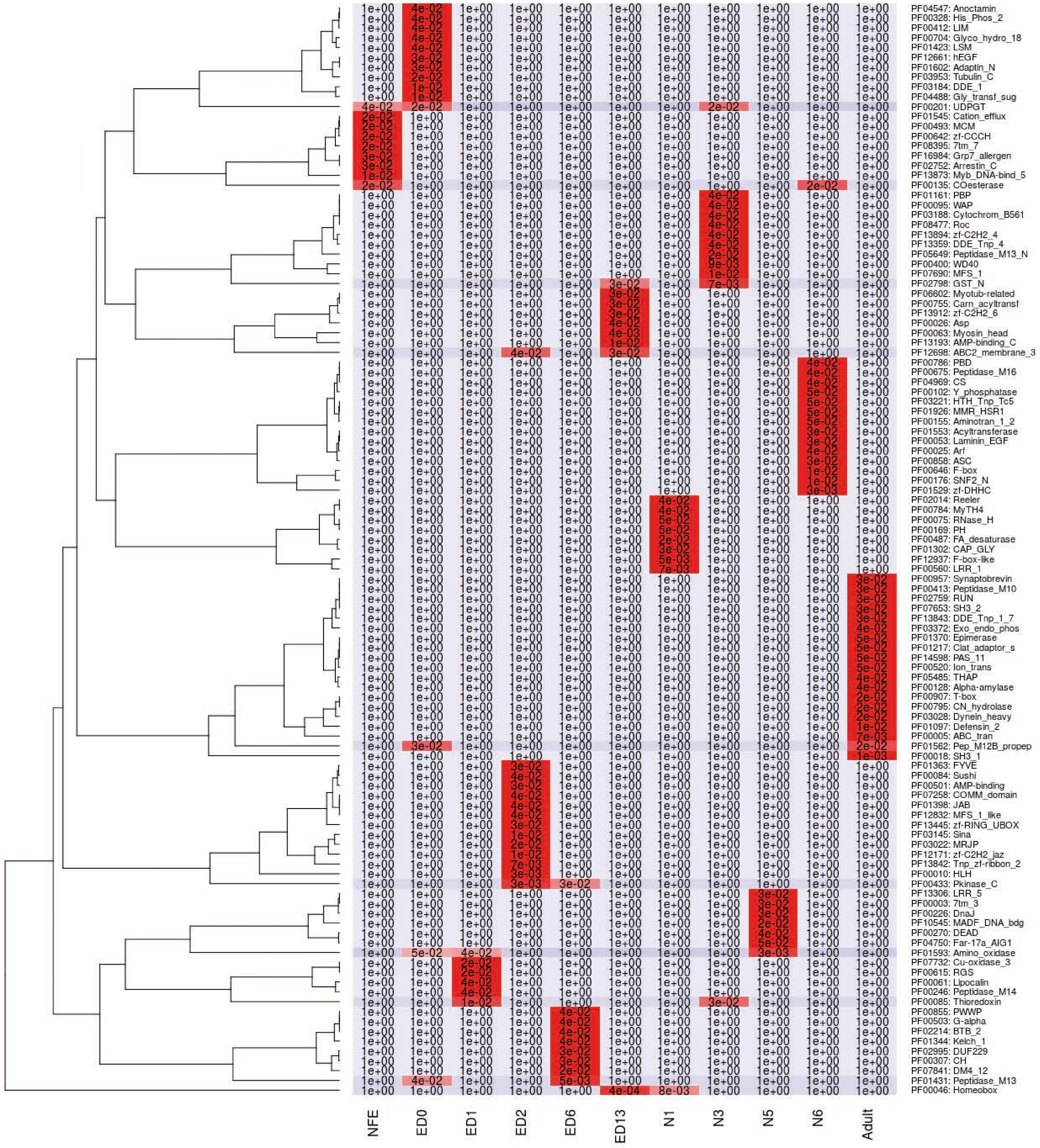

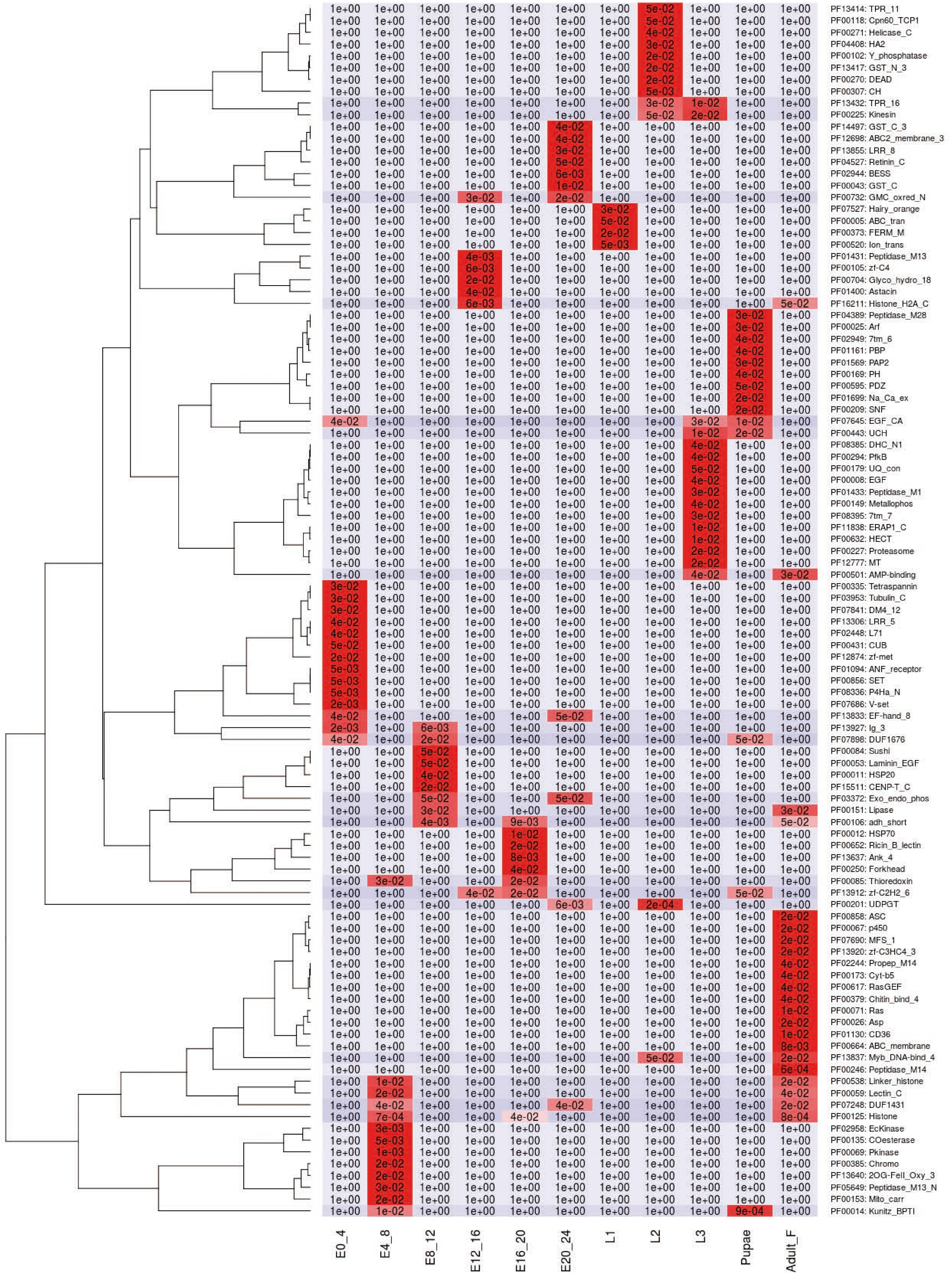

Figure S2. PFAM motifs enrichment analysis carried out in each stage of of *Blattella germanica* (left) and *Drosophila melanogaster* (right). Related to Figure 2B. Only genes whose expression was >1 FPKM were considered. The p-value of the hypergeometric test is shown, and only those PFAMs showing a significant enrichment (p-value < 0.05), in at least one stage, are shown. The color scale from red (low p-value) to blue (high p-value) is normalized at each row.

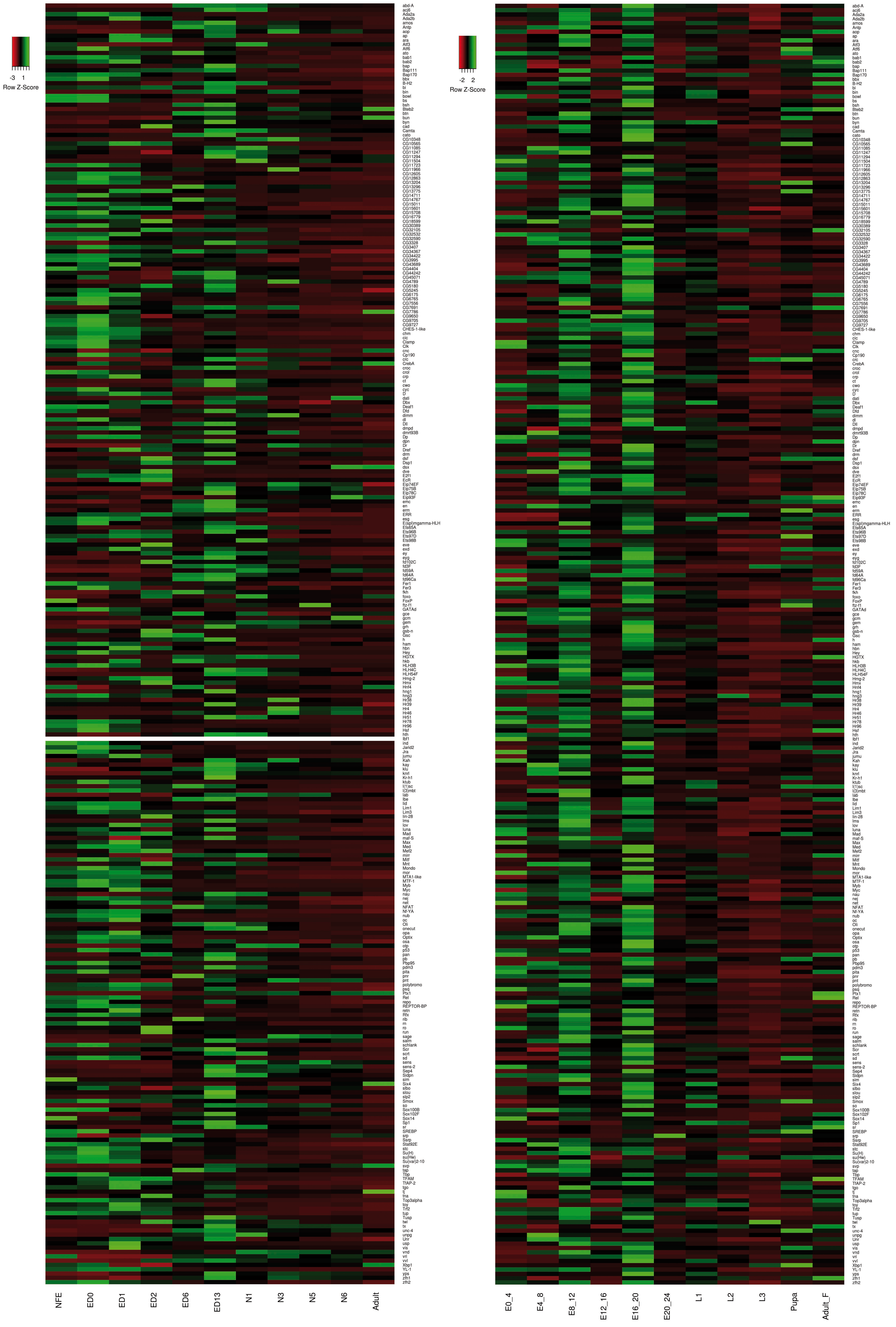

Figure S3. Expression in *Blattella germanica* (left) and *Drosophila melanogaster* (right) of the 297 transcription factors common to the two species. Related to Figure 5A.

Table S1. Biological data corresponding to the 11 transcriptomes studied in the present work. Related to Experimental Procedures. Data on juvenile hormone (JH) and ecdysteroids (20E) are from Treiblmayr et al. (2006) (JH in nymphal stages), Maestro et al. (2010) (JH in embryo stages), Cruz et al. (2003) (20E in nymphal stages), Piulachs et al. (2010) (20E in embryo stages). Stages are from Tanaka (1976). Scale bars equivalent to 500 µM (from NFE to ED13) or to 1 mm (from N1 to adult).

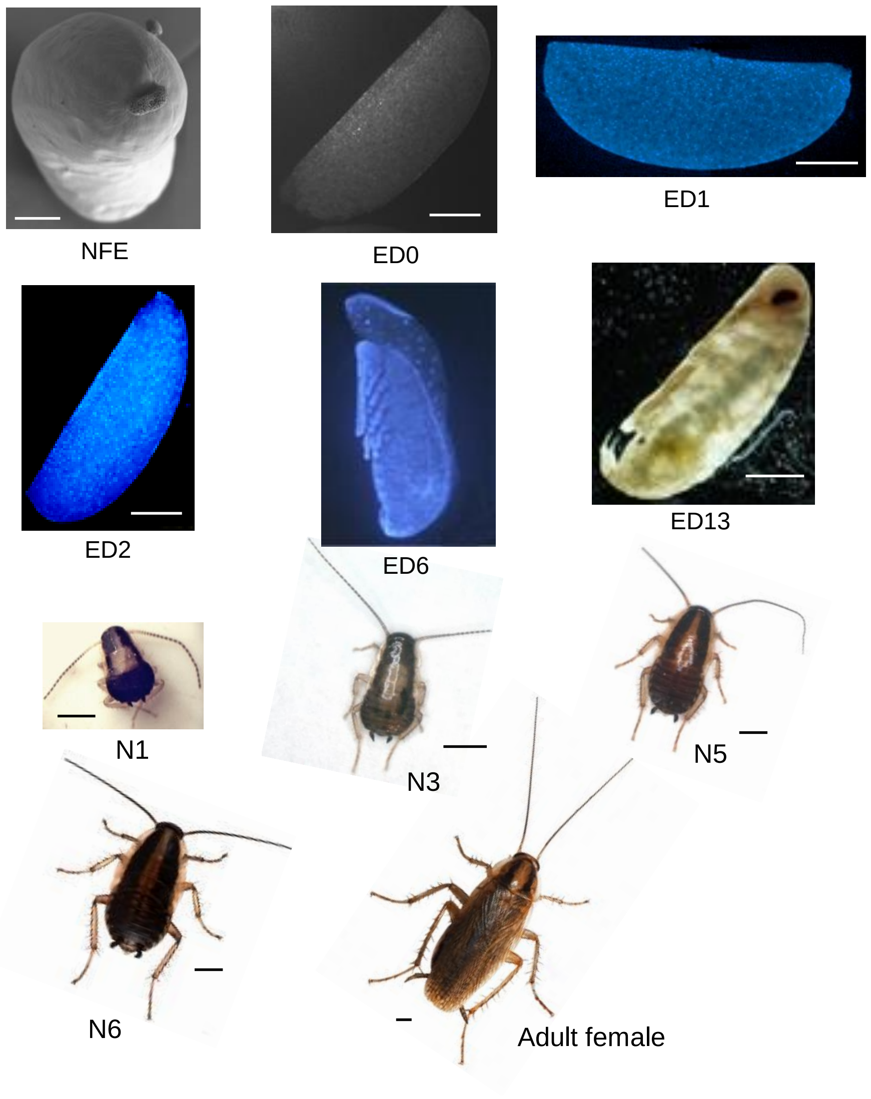

Library	Period development	Pooled individuals per sample (n = 2)	Age (AO: after oviposition)	% embryo development	Embryo development, Tanaka stage	Hormonal context
**NFE**	Egg	n 1 = 360 eggs from 24 females<br>n 2 = 270 eggs from 18 females	Day 8 of the first gonadotrophic cycle (in preoviposition)	--	--	Not measured in the egg. High levels of 20E and JH in the surrounding haemolymph.
**ED0**	Embryo	n 1 = 45 oothecae<br>n 2 = 24 oothecae	8 h AO, when the ootheca is still vertical	2%.	Only yolk granules observed.	No detectable levels of 20E and JH
**ED1**	Embryo	n 1 = 25 oothecae<br>n 2 = 24 oothecae	24 h AO	6%	Energids at low density spread in the yolk . Tanaka stage 1	No detectable levels of 20E and JH
**ED2**	Embryo	n 1 = 8 oothecae<br>n 2 = 8 oothecae	48 h AO	12%	Abundant energids, germ band anlage well delimited, slightly expanded at both sides. Tanaka stage 2	Burst of 20E inferred from the expression of HR3 (a 20E-dependent gene). No detectable JH
**ED6**	Embryo	n 1 = 4 oothecae<br>n 2 = 4 oothecae	144 h AO	33%	Pleuropodia well apparent, legs segmented, caudal space arises. Tanaka stage 8	Peak of 20E. Very low levels of JH
**ED13**	Embryo	n 1 = 4 oothecae<br>n 2 = 4 oothecae	312 h AO	72%	Eyes colored, antennae and legs reaching the 5th abdominal segment. Tanaka stage 15	Peak of 20E. High levels of JH
**N1**	1st nymphal instar	n 1 = 5 individuals (indeterminate sex)<br>n 2 = 5 individuals (indeterminate sex)	1-2 days old	--	--	High levels of 20E and JH
**N3**	3rd nymphal instar	n 1 = 5 individuals (indeterminate sex)<br>n 2 = 5 individuals (indeterminate sex)	2-4 days old	--	--	High levels of 20E and JH
**N5**	5th nymphal instar	n 1 = 5 individuals (females)<br>n 2 = 5 individuals (females)	3-5 days old	--	--	High levels of 20E and JH
**N6**	6th nymphal instar	n 1 = 5 individuals (females)<br>n 2 = 5 individuals (females)	5-7 days old	--	--	High levels of 20E, no JH
**Adult**	Adult	n 1 = 5 females<br>n 2 = 5 females	5 days old	--	--	Low (ovarian) levels of 20E and high JH levels

Table S2. Caracteristics of the embryo mRNA libraries of *Blattella germanica* and *Drosophila melanogaster* used in this work. Related to Experimental Procedures. Same color indicates equivalent developmental periods.

Blattella germanica		***Drosophila melanogaster***	
**Library, hours and developmental/molecular features**	**% development (a)**	**Library, hours and developmental/molecular features**	**% development (b)**
NFE-Non-fecunded eggs	0.00%		
ED0 8h AO. Pre-blastoderm stage. T48 highly expressed. Maternal-gap genes (Nanos and tailless) well expressed.	2.00%	Embryo 0-4h. Pre-bastoderm+blastoderm+gastrula stages. T48 highly expressed. Maternal-gap genes (Nanos, hunchback, caudal and tailless) well expressed.	0.0% - 17%
ED1 24h AO. Early blastoderm. T48 still expressed. Gap genes (orthodenticle, huckbein) well expressed. Pair rule genes not expressed yet.	6.00%	Embryo 4-8h. Early extended germ band+late extended germ band. Gap, pair rule and segmentation genes well expressed.	17% - 34%
ED2 48h AO. Germ band being formed. Pair rule and segmentation genes well expressed.	12.00%	Embryo 8-12h. Onset of dorsal closure. Gap, pair rule and segmentation genes well expressed. Hox genes expressed.	34% - 50%
ED6 144h AO. Onset of dorsal closure. Segmentation genes still expressed. Hox genes begin to be expressed.	33.00%	Embryo 12-16h. End of dorsal closure+beginning of late embryo stage.	50% - 67%
ED13 312h AO. Dorsal closure finished. Hox genes well expressed.	72.00%	Embryo_16-20h. Late embryo stage. Hox genes well expressed.	67% - 83%
		Embryo 20-24h. Late embryo stage.	83% - 100%

(a) 100% = 18 days
(b) 100% = 24 hours

Table S3. Names and the corresponding accession codes of genes whose expression profiles are showed. Related to Experimental Procedures. For *Drosophila melanogaster* the Flybase gene accession is used and for *Blattella germanica*, the codes were based on genome annotation available from NCBI bioproject under the accession code PRJNA427252.

Gene name	FlyBase Accession	Bger code
abdominal A	FBgn0000014	Bger_24605
Abdominal B	FBgn0000015	BgerTmpA028008-RA[a]
ACAT	FBgn0035203	Bger_10151[b]
ALDH	FBgn0010548	Bger_14856[c]
antennapedia	FBgn0260642	Bger_28331
anterior open	FBgn0000097	Bger_12886
apterous	FBgn0267978	Bger_ M0006[a]
bicoid	FBgn0000166	No orthologous in *B. germanica*
brachyenteron	FBgn0011723	Bger_17804
Broad Complex (Core)	FBgn0283451	Bger_M0004[a]
buttonless	FBgn0014949	Bger_04705
CAMTA	FBgn0259234	Bger_11477
caudal	FBgn0000251	Bger_M0002[a]
collier	FBgn0001319	Bger_28042
crocodile	FBgn0014143	Bger_08908
crooked legs	FBgn0020309	Bger_24171
cropped	FBgn0001994	Bger_15672
deadpan	FBgn0010109	Bger_12374
Deaf1	FBgn0013799	Bger_03953
defective proventriculus	FBgn0020307	Bger_25497
Deformed	FBgn0000439	Bger_21960
Disembodied	FBgn0000449	Bger_24331
Dnmt1	No orthologous in *D. melanogaster*	Bger_02650
Dnmt2	FBgn0028707	Bger_06901
E(spl)mgamma-HLH	FBgn0002735	Bger_05984
E75	FBgn0000568	Bger_26014
E93	FBgn0264490	Bger_M0005[a]
EcR	FBgn0000546	Bger_08790
empty-spiracles	FBgn0000576	Bger_26557
engrailed	FBgn0000577	Bger_00720
engrailed	FBgn0000577	Bger_00720
estrogen-related receptor	FBgn0035849	Bger_11720
Ets at 65A	FBgn0005658	Bger_27146
even skipped	FBgn0000606	Bger_18070
FAMeT	FBgn0034583	Bger_20710[c]
FPPS	FBgn0025373	Bger_01636[b]
Ftz-f1	FBgn0001078	Bger_09989
fushitarazu	FBgn0001077	Bger_21966
gce	FBgn0261703	No orthologous in *B. germanica*
glial cells missing	FBgn0014179	Bger_00054
grainy head	FBgn0259211	Bger_26553
hairy	FBgn0001168	Bger_26281
hedgehog	FBgn0004644	Bger_02233
Helix loop helix protein 3B	FBgn0011276	Bger_13620
HMGR	FBgn0263782	Bger_14007[b]
HMGS1	No orthologous in *D. melanogaster*	Bger_03932[b]
HMGS2	FBgn0010611	Bger_02739[b]
HR3	FBgn0000448	Bger_00728

HR4	FBgn0264562	Bger_18448
hunchback	FBgn0001180	Bger_14305
IPPI	FBgn0038876	Bger_04562[b]
JHAMT	FBgn0028841	Bger_04116[c]
kirps	FBgn0001320	Bger_03424
krüppel	FBgn0001325	Bger_01853
Krüppel homolog 1	FBgn0266450	Bger_M0003[a]
labial	FBgn0002522	Bger_17181
lateral muscle scarcer	FBgn0002023	Bger_02888
lethal of scute	FBgn0002561	Bger_14544
little imaginal disc	FBgn0031759	Bger_06256
Met	FBgn0002723	Bger_10267
MVD	FBgn0030683	Bger_12150[b]
MvK	FBgn0061359	Bger_05361[b]
myo	FBgn0026199	Bger_15600
nanos	FBgn0002962	Bger_23144
Neverland	FBgn0259697	Bger_22030
orthodenticale	FBgn0004102	Scaffold533:124865-197312[a]
orthopedia	FBgn0015524	Bger_23610
Phantom	FBgn0004959	Bger_21433
Pmvk	FBgn0032811	Bger_06941[b]
pou domain motif 3	FBgn0261588	Bger_09030
proboscipedia	FBgn0051481	Bger_17179
Relish	FBgn0014018	Bger_13050
ribbon	FBgn0003254	Bger_21758
runt	FBgn0003300	Bger_05999
senseless	FBgn0051632	Bger_16235
sex combs reduced	FBgn0003339	Bger_21963
Shade	FBgn0003388	Bger_13798
Shadow	FBgn0003312	Bger_09617
Shroud	FBgn0262112	Bger_07744
shuttle craft	FBgn0001978	Bger_06059
smaug	FBgn0016070	Bger_10865
Sox box protein 14	FBgn0005612	Bger_02065
Spook	FBgn0003486	Bger_25648
Spookiest	No orthologous in *D. melanogaster*	Bger_04901
SREBP	FBgn0261283	Bger_16229
suppressor of Hairy wing	FBgn0003567	Bger_00591
tailless	FBgn0003720	Bger_19904
Taiman	FBgn0041092	Bger_21670
ultrabithorax	FBgn0003944	BgerTmpA017800-RA[a]
unplugged	FBgn0015561	Bger_08368
USP	FBgn0003964	Bger_15477[b]
vismay	FBgn0033748	Bger_07739
wingless	FBgn0004009	Bger_10842
zelda	FBgn0259789	Bger_M0001[a]

[a] Genes manually annotated in *B. germanica* genome.

[b] Annotation based on Qu et al. (2017). MicroRNAs regulate the sesquiterpenoid hormonal pathway in *Drosophila* and other arthropods. Proceedings of the Royal Society B: Biological Sciences 284(1869). pii: 20171827.

[c] Annotation based on Harrison et al. (2018). Hemimetabolous genomes reveal molecular basis of termite eusociality. Nature Ecology and Evolution 2(3):557–566.

Transparent Methods

Insects

B. germanica specimens were obtained from a colony reared in the dark at 29 ± 1ºC and 60-70% relative humidity. All dissections and tissue sampling were carried out on carbon dioxide-anesthetized specimens. Tissues were frozen on liquid nitrogen and stored at -80ºC until use.

Preparation and sequencing of mRNA libraries

We sequenced two biological replicates of each chosen stage along the ontogeny of the cockroach *B. germanica* (Table S1). Data on JH and 20E for the chosen stages are from Treiblmayr et al. (2006) (JH in nymphal stages), Maestro et al. (2010) (JH in embryo stages), Cruz et al. (2003) (20E in nymphal stages) and Piulachs et al. (2010) (20E in embryo stages). Tanaka stages are from Tanaka (1976). Total RNA was extracted using the GenElute Mammalian Total RNA kit (Sigma) following the manufacturer's protocol. Up to 10 µg of total RNA from pooled samples were used to prepare the libraries. The mRNAs were isolated by magnetic beads using the Dynabeads® Oligo (dT)25 (Invitrogen, Life Technologies) and following the manufacturer's protocol. Quality and quantity of mRNAs were assessed with a Bioanalyzer (Aligent Bioanalyzer® 2100). Libraries were prepared using NEBNext mRNA library Prep Master Mix Set for Illumina sequencing (New England Biolabs), and sequenced with 6 multiplexed runs of Illumina MiSeq. We did paired-end sequencing, with read length of 300 nucleotides. To avoid batch effects, replicates were never multiplexed together in the same run. We made all the datasets publicly available at Gene Expression Omnibus (Edgar et al. 2002) under the accession code GEO: GSE99785. For comparisons, we used an equivalent RNA-seq dataset of *D. melanogaster* (GEO: GSE18068) comprises 22 libraries from 11 developmental stages (2 replicates each) covering the entire embryo development, the three larval stages, the pupa, and the adult female. In postembryonic stages we followed the correspondence *B. germanica* pre-last nymphal instars with *D. melanogaster* larvae, the last nymphal instar with the pupa (Belles and Santos 2014), and the respective adult female stages. Correspondences between embryo stages of *D. melanogaster* and *B. germanica* are summarized in Table S2.

Analysis of the RNA libraries

In the *B. germanica* libraries, we removed the adapters and trimmed the low quality bases on the reads extremes using Trimmomatic (version 0.32, relevant parameters: ILLUMINACLIP:"/TruSeq3-PE-2.fa":2:30:10:8:TRUE SLIDINGWINDOW:4:15) (Bolger et al. 2014). RNA-seq data along the development of *D. melanogaster* was retrieved from Gene Expression Omnibus under the accession GSE18068. All the RNA-seq datasets, were mapped to their correspondent insect genome using the STAR software (version 2.3.0, using default parameters) (Dobin et al. 2013) and the table of counts obtained with the R implementation of featureCounts (version 1.22.3, relevant parameters: allowMultiOverlap=T, countMultiMappingReads=T, useMetaFeatures=T) (Liao et al. 2014), using the corresponding gene annotation of each insect (Dataset S2 and S3). The genome assembly of *B. germanica* and corresponding gene annotations are available from NCBI bioproject under the accession code PRJNA427252. Regarding *D. melanogaster*, we used the genome assembly and gene annotation version "dmel_r6.12", available in Flybase (http://flybase.org/). For clustering purposes reads were normalized with the "varianceStabilizingTransformation" function implemented at DESeq2 R package (version 1.12.4) (Love et al. 2014), for gene expression profiles and visualization (e.g. heatmaps and bar plots) we used the FPKM normalization.

Functional annotation of genes

Using the protein sequence, functional annotation was obtained using PfamScan (version 1.5, Database Pfam-A, release 30.0) (Bateman et al. 2004; Li et al. 2015). Then, we selected those genes with a Pfam motifs unequivocally related to TF activity (de Mendoza et al. 2013; Ylla and Belles 2015). GO-terms were retrieved for the *D. melanogaster* genes with the AnnotationForge package (version 1.14.2) (Carlson and Pages 2016), and used for the corresponding *B. germanica* orthologues. Orthologous genes shared by *B. germanica* and *D. melanogaster* where obtained by following the Blastp (version 2.5.0+) (Camacho et al. 2009) reciprocal best hits (BRBHs) strategy (Rivera et al. 1998) (Dataset S4). For the Hox genes, we aligned the candidate of *B. germanica* protein sequences with the eight canonical Hox genes (Negre and Ruiz 2007) of different insect species with ClustalX (Larkin et al. 2007). Then, we performed a phylogenetic reconstruction with RAxML (within CIPRESScience Gateway, version V8.2X) (Stamatakis 2014), which is based on the maximum-likelihood principle, a JTT matrix, a gamma model of heterogeneity rate, and using empirical base frequencies and estimating proportions. The data was bootstrapped for 100 replicates. The accession

codes in both insects of these Hox genes, and that of other manually curated orthologous genes, are detailed in Table S3. The enrichments analysis tests for GO-terms was performed on the subset of expressed genes at each stage (>1FPKM) using the hypergeometrical test implemented in the GOstats package (version 2.38.1, relevant parameters: ontology = biologica process) (Falcon and Gentleman 2007), while the enrichment analysis for Pfam motifs was done on the same subset of genes with the hypergeometric test implemented in R (relevant parameters:phyper()) (R Development Core Team 2011).

Differential expression analysis

The differential expression analyses tests were performed with all genes using the DESeq2 package (Love et al. 2014). The obtained P-values were adjusted for multiple testing using the FDR (False Discovery Rate), and the threshold for significant expression change was set at an adjusted p-value < 0.05.

DNA methylation

We calculated the ratio between the observed frequency of CpG and the expected frequency in the gene body of each annotated gene (Elango et al. 2009).

$$\mathrm{CpGo/e} = \frac{f(CG)}{f(C) * f(G)}$$

The regression between $\mathrm{CpG}_{o/e}$ of each gene and their expression level at each library was tested in R using the Pearson's product moment correlation coefficient.

Quantification of mRNA levels of Zelda by qRT-PCR

Quantitative real-time PCR (qRT-PCR) was carried out in an iQ5 Real-Time PCR Detection System (Bio-Lab Laboratories), using SYBR®Green (iTaq ™Universal SYBR® Green Supermix; Applied Biosystems). Reactions were triplicate, and a template-free control was included in all batches. Primers used to detect Zelda mRNA levels were: TGTCCCAAACAGTTCAACCA (forward) and AAAGGGTTTCTCTCCCGTGT (reverse) designed on the sequence deposited in GenBank under the accession code LT717628.1. We validated the efficiency of each set

of primers by constructing a standard curve through three serial dilutions. In all cases, levels of mRNA were calculated relative to BgActin-5c mRNA levels,which were measured using the primers AGCTTCCTGATGGTCAGGTGA (forward) and TGTCGGCAATTCCAGGGTACATGGT (reverse), based on the sequence with the GenBank accession code AJ862721. Three biological replicates per point were measured and averaged, and results were calculated as copies of Zelda mRNA per 100 copies of BgActin-5c mRNA.

Phylogenetic analysis of Hox proteins

Sequences used were obtained by Blast from GenBank or from i5k project (https://i5k.nal.usda.gov/webapp/blast/)andfromFlybase(http://flybase.org/). Alignments were carried out with ClustalX (Larkin et al. 2007) and phylogenetic reconstruction with RAxML (Stamatakis 2014), based on the maximum-likelihood principle, a JTT matrix, a gamma model of heterogeneity rate, and using empirical base frequencies and estimating proportions. The data was bootstrapped for 100 replicates. The sequences used for comparison with those of *Blattella germanica* were: *Acromyrmex echinatior* EGI64564.1 (proboscipedia, pb); *Anopheles gambiae* XP_311623.2 (Ultrabithorax, Ubx); *Anoplophora glabripennis* XP_018562491 (Sex combs reduced, Scr); *Biston betularia* ADO33070.2 (Ubx); *Bombyx mori* NP_001107632.1 (Ubx); *Callimorpha dominula* AIB07881.1 (Deformed, Dfd); *Camponotus floridanus* EFN67233.1 (abdominal-A, abd-A); *Drosophila melanogaster* FBgn0000014 (abd-A),NP_524896 (Abdominal-B, Abd-B), FBgn0260642 (Antennapedia, Antp), FBgn0000439 (Dfd), FBgn0002522 (labial, lab),FBgn0051481(pb), FBgn0003339 (Scr), FBpp0082793 (Ubx); *Harpegnathos saltator* XP_011148886.1 (abd-A), EFN88927.1 (Scr); *Lasius niger* MQ93049.1 (lab); *Lucilia cuprina* KNC34760.1 (Antp); *Megachile rotundata* XP_012154280.1 (lab); *Operophtera brumata* KOB75113.1 (Abd-B); *Periplaneta americana* ADF35697.1 (Scr); *Schistocerca americana* AAB03236.1 (Antp); *Tribolium castaneum* AAB70263.1 (abd-A), NP_001034519.1 (Abd-B), NP_001107762.1 (lab), AAF03888.1 (pb), AAG13009.1 (Scr); *Zootermopsis nevadensis* KDR16991.1 (abd-A), KDR11585.1 (Antp), XP_021919824.1 (Dfd), KDR19418.1 (lab), KDR19417.1 (pb), KDR19415.1 (Scr).

References

Bateman, A., Coin, L., Durbin, R., Finn, R. D., Hollich, V., Griffiths-Jones, S., Khanna, A., Marshall, M., Moxon, S., Sonnhammer, E. L. L., et al. (2004). The Pfam protein families database. Nucleic Acids Res. 32, D138-41.

Belles, X. and Santos, C. G. (2014). The MEKRE93 (Methoprene tolerant-Krüppel homolog 1-E93) pathway in the regulation of insect metamorphosis, and the homology of the pupal stage. Insect Biochem. Mol. Biol. 52, 60–68.

Bolger, A. M., Lohse, M. and Usadel, B. (2014). Trimmomatic: a flexible trimmer for Illumina sequence data. Bioinformatics 30, btu170-.

Camacho, C., Coulouris, G., Avagyan, V., Ma, N., Papadopoulos, J., Bealer, K. and Madden, T. L. (2009). BLAST+: architecture and applications. BMC Bioinformatics 10, 421.

Carlson, M. and Pages, H. (2016). AnnotationForge: Code for Building Annotation Database Packages. Available online at https://bioconductor.org/packages/release/bioc/html/AnnotationForge.html/.

Cruz, J., Martín, D., Pascual, N., Maestro, J. L., Piulachs, M. D. and Bellés, X. (2003). Quantity does matter. Juvenile hormone and the onset of vitellogenesis in the German cockroach. Insect Biochem. Mol. Biol. 33, 1219–1225.

Dobin, A., Davis, C. A., Schlesinger, F., Drenkow, J., Zaleski, C., Jha, S., Batut, P., Chaisson, M. and Gingeras, T. R. (2013). STAR: ultrafast universal RNA-seq aligner. Bioinformatics 29, 15–21.

Edgar, R., Domrachev, M. and Lash, A. E. (2002). Gene Expression Omnibus: NCBI gene expression and hybridization array data repository. Nucleic Acids Res. 30, 207–210.

Elango, N., Hunt, B. G., Goodisman, M. A. D. and Yi, S. V (2009). DNA methylation is widespread and associated with differential gene expression in castes of the honeybee, *Apis mellifera*. Proc. Natl. Acad. Sci. U. S. A. 106, 11206–11211.

Falcon, S. and Gentleman, R. (2007). Using GOstats to test gene lists for GO term association. Bioinformatics 23, 257–258.

Larkin, M. A., Blackshields, G., Brown, N. P., Chenna, R., McGettigan, P. A., McWilliam, H., Valentin, F., Wallace, I. M., Wilm, A., Lopez, R., et al. (2007). Clustal W and Clustal X version 2.0. Bioinformatics 23, 2947–2948.

Li, W., Cowley, A., Uludag, M., Gur, T., McWilliam, H., Squizzato, S., Park, Y. M., Buso, N. and Lopez, R. (2015). The EMBL-EBI bioinformatics web and programmatic tools framework. Nucleic Acids Res. 43, W580-4.

Liao, Y., Smyth, G. K. and Shi, W. (2014). featureCounts: an efficient general purpose program for assigning sequence reads to genomic features. Bioinformatics 30, 923–930.

Love, M. I., Huber, W. and Anders, S. (2014). Moderated estimation of fold change and dispersion for RNA-seq data with DESeq2. Genome Biol. 15, 550.

Maestro, J. L., Pascual, N., Treiblmayr, K., Lozano, J. and Belles, X. (2010). Juvenile hormone and allatostatins in the German cockroach embryo. Insect Biochem. Mol. Biol. 40, 660–665.

de Mendoza, A., Sebé-Pedrós, A., Sestak, M. S., Matejcic, M., Torruella, G., Domazet-Loso, T. and Ruiz-Trillo, I. (2013). Transcription factor evolution in eukaryotes and the assembly of the regulatory toolkit in multicellular lineages. Proc. Natl. Acad. Sci. U. S. A. 110, E4858-4866.

Negre, B. and Ruiz, A. (2007). HOM-C evolution in *Drosophila*: is there a need for Hox gene clustering? Trends Genet. 23, 55-59.

Piulachs, M.-D., Pagone, V. and Belles, X. (2010). Key roles of the Broad-Complex gene in insect embryogenesis. Insect Biochem. Mol. Biol. 40, 468–475.

R Development Core Team (2011). R: A Language and Environment for Statistical Computing. Vienna, Austria : the R Foundation for Statistical Computing. ISBN: 3-900051-07-0. Available online at http://www.R-project.org/.

Rivera, M. C., Jain, R., Moore, J. E. and Lake, J. A. (1998). Genomic evidence for two functionally distinct gene classes. Proc. Natl. Acad. Sci. U. S. A. 95, 6239–6244.

Stamatakis, A. (2014). RAxML version 8: a tool for phylogenetic analysis and post-analysis of large phylogenies. Bioinformatics 30, 1312–1313.

Tanaka, A. (1976). Stages in the embryonic development of the German cockroach, *Blattella germanica* Linné (Blattaria, Blattellidae). Kontyû, Tokyo 44, 1703–1714.

Treiblmayr, K., Pascual, N., Piulachs, M.-D. M. D. M.-D., Keller, T. and Belles, X. (2006). Juvenile hormone titer versus juvenile hormone synthesis in female nymphs and adults of the German cockroach, *Blattella germanica*. J. Insect Sci. 6, 1–7.

Ylla, G. and Belles, X. (2015). Towards understanding the molecular basis of cockroach tergal gland morphogenesis. A transcriptomic approach. Insect Biochem. Mol. Biol. 63, 104–112.
